# Supplementary material for: CXR-LT 2024: A MICCAI challenge on long-tailed, multi-label, and zero-shot disease classification from chest X-ray
Source: ArXiv. 2025 Jun 9:arXiv:2506.07984v1. Preprint. [Version 1] (PMC12306832)
Supplement: 1 [file NIHPP2506.07984V1-supplement-1.pdf]

Supplementary Material

CXR-LT 2024: A MICCAI challenge on long-tailed, multi-label, and zero-shot disease classification from chest X-ray

Supplementary Table 1. Final test phase results of the CXR-LT 2024 competition for Task 1. The table presents the precision of the top-4 teams' final models for each of the 40 classes evaluated on the test set.

Disease	zguo	Tianjie_dai	XYPB	dongkyunk
Atelectasis	0.626	0.630	0.627	0.628
Cardiomegaly	0.669	0.683	0.678	0.675
Consolidation	0.250	0.259	0.253	0.251
Edema	0.551	0.551	0.553	0.547
Enlarged Cardiomediastinum	0.189	0.196	0.192	0.188
Fracture	0.380	0.364	0.328	0.367
Lung Lesion	0.068	0.069	0.067	0.062
Lung Opacity	0.628	0.627	0.629	0.633
Pleural Effusion	0.845	0.845	0.843	0.842
Pneumonia	0.335	0.336	0.342	0.339
Pneumothorax	0.608	0.597	0.597	0.618
Support Devices	0.927	0.925	0.927	0.926
Granuloma	0.317	0.269	0.296	0.342
Normal	0.331	0.333	0.335	0.334
Calcification of the Aorta	0.172	0.163	0.164	0.173
Emphysema	0.263	0.262	0.258	0.274
Fibrosis	0.169	0.167	0.169	0.138
Hernia	0.572	0.583	0.580	0.550
Infiltration	0.064	0.064	0.067	0.064
Mass	0.251	0.287	0.250	0.258
Nodule	0.246	0.233	0.232	0.254
Pleural Thickening	0.153	0.146	0.138	0.137
Pneumomediastinum	0.307	0.340	0.338	0.300
Pneumoperitoneum	0.299	0.299	0.351	0.286
Subcutaneous Emphysema	0.613	0.604	0.580	0.609
Tortuous Aorta	0.067	0.068	0.070	0.072
Adenopathy	0.103	0.136	0.129	0.112
Azygos Lobe	0.156	0.133	0.117	0.146
Clavicle Fracture	0.040	0.013	0.006	0.003
Fissure	0.223	0.198	0.174	0.197
Hydropneumothorax	0.179	0.185	0.173	0.142
Infarction	0.011	0.008	0.011	0.011
Kyphosis	0.086	0.086	0.107	0.073
Lobar Atelectasis	0.008	0.004	0.005	0.004
Pleural Other	0.077	0.066	0.067	0.071
Pulmonary Embolism	0.012	0.013	0.013	0.012
Pulmonary Hypertension	0.032	0.047	0.049	0.035
Rib Fracture	0.284	0.269	0.256	0.289
Round(ed) Atelectasis	0.053	0.028	0.057	0.041
Tuberculosis	0.062	0.060	0.073	0.064
Mean	0.281	0.279	0.277	0.277

Supplementary Table 2. Final test phase results of the CXR-LT 2024 competition for Task 1. The table presents the F1 of the top-4 teams' final models for each of the 40 classes evaluated on the test set.

Disease	zguo	Tianjie_dai	XYPB	dongkyunk
Atelectasis	0.566	0.549	0.557	0.556
Cardiomegaly	0.541	0.517	0.543	0.543
Consolidation	0.259	0.275	0.275	0.275
Edema	0.453	0.440	0.468	0.455
Enlarged Cardiomediastinum	0.232	0.220	0.229	0.229
Fracture	0.347	0.320	0.359	0.324
Lung Lesion	0.136	0.156	0.139	0.117
Lung Opacity	0.560	0.550	0.565	0.574
Pleural Effusion	0.707	0.682	0.704	0.708
Pneumonia	0.346	0.338	0.347	0.347
Pneumothorax	0.491	0.500	0.493	0.477
Support Devices	0.841	0.813	0.841	0.845
Granuloma	0.346	0.347	0.393	0.358
Normal	0.360	0.359	0.379	0.373
Calcification of the Aorta	0.258	0.214	0.254	0.224
Emphysema	0.293	0.313	0.314	0.283
Fibrosis	0.245	0.255	0.244	0.225
Hernia	0.522	0.498	0.566	0.438
Infiltration	0.114	0.109	0.119	0.109
Mass	0.263	0.299	0.300	0.263
Nodule	0.260	0.228	0.259	0.242
Pleural Thickening	0.202	0.208	0.209	0.177
Pneumomediastinum	0.370	0.392	0.407	0.391
Pneumoperitoneum	0.444	0.443	0.481	0.386
Subcutaneous Emphysema	0.593	0.572	0.605	0.564
Tortuous Aorta	0.113	0.133	0.125	0.126
Adenopathy	0.151	0.156	0.172	0.146
Azygos Lobe	0.163	0.326	0.221	0.289
Clavicle Fracture	0.065	0.000	0.000	0.000
Fissure	0.257	0.230	0.247	0.217
Hydropneumothorax	0.313	0.270	0.258	0.231
Infarction	0.000	0.000	0.025	0.017
Kyphosis	0.167	0.123	0.125	0.129
Lobar Atelectasis	0.000	0.000	0.000	0.000
Pleural Other	0.117	0.121	0.125	0.152
Pulmonary Embolism	0.000	0.000	0.006	0.015
Pulmonary Hypertension	0.011	0.062	0.099	0.079
Rib Fracture	0.326	0.287	0.313	0.293
Round(ed) Atelectasis	0.000	0.000	0.043	0.081
Tuberculosis	0.143	0.136	0.162	0.151
Macro F1	0.289	0.286	0.299	0.285
Micro F1	0.471	0.455	0.474	0.462

Supplementary Table 3. Final test phase results of the CXR-LT 2024 competition for Task 1. The table presents the False Negative Rate (FNR) of the top-4 teams' final models for each of the 40 classes evaluated on the test set.

Disease	zguo	Tianjie_dai	XYPB	dongkyunk
Atelectasis	0.069	0.052	0.059	0.556
Cardiomegaly	0.064	0.036	0.063	0.543
Consolidation	0.271	0.305	0.307	0.275
Edema	0.121	0.108	0.131	0.455
Enlarged Cardiomediastinum	0.168	0.083	0.139	0.229
Fracture	0.362	0.394	0.438	0.324
Lung Lesion	0.851	0.814	0.761	0.117
Lung Opacity	0.042	0.033	0.045	0.574
Pleural Effusion	0.061	0.048	0.059	0.708
Pneumonia	0.050	0.024	0.049	0.347
Pneumothorax	0.264	0.277	0.263	0.477
Support Devices	0.055	0.049	0.056	0.845
Granuloma	0.529	0.614	0.596	0.358
Normal	0.101	0.101	0.130	0.373
Calcification of the Aorta	0.468	0.331	0.420	0.224
Emphysema	0.440	0.467	0.472	0.283
Fibrosis	0.509	0.512	0.545	0.225
Hernia	0.379	0.329	0.378	0.438
Infiltration	0.802	0.854	0.768	0.109
Mass	0.560	0.530	0.621	0.263
Nodule	0.568	0.474	0.571	0.242
Pleural Thickening	0.554	0.557	0.679	0.177
Pneumomediastinum	0.497	0.527	0.510	0.391
Pneumoperitoneum	0.597	0.551	0.473	0.386
Subcutaneous Emphysema	0.109	0.087	0.127	0.564
Tortuous Aorta	0.836	0.713	0.784	0.126
Adenopathy	0.819	0.675	0.748	0.146
Azygos Lobe	0.849	0.472	0.660	0.289
Clavicle Fracture	0.958	1.000	1.000	0.000
Fissure	0.541	0.519	0.645	0.217
Hydropneumothorax	0.636	0.594	0.693	0.231
Infarction	1.000	1.000	0.986	0.017
Kyphosis	0.703	0.533	0.713	0.129
Lobar Atelectasis	1.000	1.000	1.000	0.000
Pleural Other	0.913	0.889	0.858	0.152
Pulmonary Embolism	1.000	1.000	0.996	0.015
Pulmonary Hypertension	0.994	0.958	0.891	0.079
Rib Fracture	0.499	0.517	0.526	0.293
Round(ed) Atelectasis	1.000	1.000	0.971	0.081
Tuberculosis	0.759	0.756	0.665	0.151
Macro FNR	0.525	0.495	0.520	0.466
Micro FNR	0.151	0.135	0.153	0.142

Supplementary Table 4. Final test phase results of the CXR-LT 2024 competition for Task 2. The table presents the precision of the top-4 teams' final models for each of the 26 classes evaluated on the golden standard test set.

Disease	XYPB	zguo	yangz16	YYama
Atelectasis	0.464	0.457	0.454	0.465
Calcification of the Aorta	0.680	0.643	0.566	0.633
Cardiomegaly	0.747	0.711	0.717	0.739
Consolidation	0.466	0.437	0.450	0.466
Edema	0.537	0.582	0.564	0.585
Emphysema	0.364	0.386	0.379	0.335
Enlarged Cardiomediastinum	0.347	0.346	0.348	0.339
Fibrosis	0.549	0.456	0.543	0.536
Fracture	0.538	0.622	0.619	0.492
Hernia	0.789	0.748	0.840	0.741
Infiltration	0.052	0.049	0.056	0.045
Lung Lesion	0.037	0.048	0.055	0.042
Lung Opacity	0.657	0.658	0.642	0.662
Mass	0.430	0.388	0.384	0.401
Normal	0.726	0.795	0.741	0.727
Nodule	0.326	0.430	0.335	0.376
Pleural Effusion	0.837	0.850	0.842	0.835
Pleural Other	0.339	0.262	0.259	0.307
Pleural Thickening	0.333	0.321	0.372	0.258
Pneumomediastinum	0.832	0.791	0.768	0.823
Pneumonia	0.127	0.165	0.125	0.149
Pneumoperitoneum	0.732	0.605	0.641	0.581
Pneumothorax	0.665	0.657	0.630	0.639
Subcutaneous Emphysema	0.849	0.802	0.760	0.783
Support Devices: 0.9643	0.964	0.961	0.944	0.958
Tortuous Aorta	0.302	0.319	0.245	0.317
Mean	0.527	0.519	0.511	0.509

Supplementary Table 5. Final test phase results of the CXR-LT 2024 competition for Task 2. The table presents the F1 of the top-4 teams' final models for each of the 26 classes evaluated on the golden standard test set.

Disease	XYPB	zguo	yangz16	YYama
Atelectasis	0.517	0.522	0.486	0.523
Calcification of the Aorta	0.628	0.543	0.000	0.548
Cardiomegaly	0.618	0.625	0.603	0.619
Consolidation	0.439	0.432	0.053	0.432
Edema	0.547	0.530	0.413	0.545
Emphysema	0.321	0.414	0.000	0.274
Enlarged Cardiomediastinum	0.437	0.415	0.032	0.433
Fibrosis	0.546	0.455	0.000	0.526
Fracture	0.552	0.583	0.154	0.544
Hernia	0.737	0.684	0.690	0.743
Infiltration	0.094	0.042	0.000	0.000
Lung Lesion	0.048	0.000	0.000	0.000
Lung Opacity	0.684	0.685	0.625	0.685
Mass	0.426	0.358	0.261	0.415
Normal	0.737	0.673	0.053	0.646
Nodule	0.364	0.375	0.000	0.310
Pleural Effusion	0.714	0.721	0.745	0.714
Pleural Other	0.240	0.000	0.000	0.095
Pleural Thickening	0.383	0.339	0.000	0.391
Pneumomediastinum	0.767	0.789	0.326	0.776
Pneumonia	0.114	0.112	0.000	0.118
Pneumoperitoneum	0.681	0.579	0.222	0.585
Pneumothorax	0.513	0.545	0.619	0.539
Subcutaneous Emphysema	0.808	0.808	0.720	0.842
Support Devices: 0.9643	0.877	0.875	0.884	0.873
Tortuous Aorta	0.174	0.150	0.000	0.143
Macro F1	0.499	0.471	0.260	0.474
Micro F1	0.564	0.562	0.530	0.562

Supplementary Table 6. Final test phase results of the CXR-LT 2024 competition for Task 2. The table presents the False Negative Rate (FNR) of the top-4 teams' final models for each of the 26 classes evaluated on the golden standard test set.

Disease	XYPB	zguo	yangz16	YYama
Atelectasis	0.016	0.032	0.456	0.016
Calcification of the Aorta	0.426	0.532	1.000	0.511
Cardiomegaly	0.019	0.025	0.484	0.025
Consolidation	0.176	0.189	0.973	0.203
Edema	0.040	0.040	0.683	0.040
Emphysema	0.552	0.379	1.000	0.655
Enlarged Cardiomediastinum	0.246	0.297	0.983	0.229
Fibrosis	0.455	0.545	1.000	0.545
Fracture	0.229	0.229	0.917	0.292
Hernia	0.263	0.316	0.474	0.316
Infiltration	0.750	0.917	1.000	1.000
Lung Lesion	0.833	1.000	1.000	1.000
Lung Opacity	0.010	0.010	0.367	0.015
Mass	0.350	0.400	0.850	0.450
Normal	0.054	0.081	0.973	0.135
Nodule	0.636	0.636	1.000	0.667
Pleural Effusion	0.011	0.005	0.240	0.005
Pleural Other	0.842	1.000	1.000	0.947
Pleural Thickening	0.591	0.545	1.000	0.591
Pneumomediastinum	0.200	0.200	0.800	0.257
Pneumonia	0.182	0.182	1.000	0.136
Pneumoperitoneum	0.333	0.542	0.875	0.500
Pneumothorax	0.020	0.082	0.388	0.020
Subcutaneous Emphysema	0.048	0.048	0.357	0.048
Support Devices: 0.9643	0.045	0.050	0.122	0.050
Tortuous Aorta	0.879	0.909	1.000	0.909
Macro FNR	0.316	0.365	0.767	0.368
Micro FNR	0.148	0.165	0.570	0.167

Supplementary Table 7. Final test phase results of the CXR-LT 2024 competition for Task 3. The table presents the precision of the top-3 teams' final models for each of the 5 classes evaluated on the test set.

Disease	yyge	zhangRuichi	pamessina
Bulla	0.013	0.011	0.038
Cardiomyopathy	0.036	0.007	0.065
Hilum	0.359	0.389	0.252
Osteopenia	0.034	0.037	0.036
Scoliosis	0.205	0.158	0.036
Mean	0.129	0.116	0.110

Supplementary Table 8. Final test phase results of the CXR-LT 2024 competition for Task 3. The table presents the F1 of the top-3 teams' final models for each of the 5 classes evaluated on the test set.

Disease	yyge	zhangRuichi	pamessina
Bulla	0.000	0.000	0.028
Cardiomyopathy	0.062	0.000	0.026
Hilum	0.000	0.000	0.289
Osteopenia	0.061	0.000	0.019
Scoliosis	0.252	0.176	0.107
Macro F1	0.075	0.035	0.094
Micro F1	0.047	0.022	0.182

Supplementary Table 9. Final test phase results of the CXR-LT 2024 competition for Task 3. The table presents the False Negative Rate (FNR) of the top-3 teams' final models for each of the 5 classes evaluated on the test set.

Disease	yyge	zhangRuichi	pamessina
Bulla	1.000	1.000	0.260
Cardiomyopathy	0.958	1.000	0.231
Hilum	1.000	1.000	0.584
Osteopenia	0.892	1.000	0.308
Scoliosis	0.762	0.896	0.616
Macro FNR	0.923	0.979	0.400
Micro FNR	0.974	0.989	0.580
